# Patients–Physician Agreement on somatic medical treatment and lifestyle interventions in Psychiatric Inpatients

**DOI:** 10.1192/j.eurpsy.2026.10458

**Published:** 2026-07-31

**Authors:** L. Nissen, A. Løkke, J. C. Frølund, O. Hilberg, M. Højlund, P. Hjort

**Affiliations:** 1 Department of psychiatry; 2 Department of Medicine, Vejle Hospital, a part of Lillebaelt Hospital, Vejle; 3 Department of psychiatry, Mental health services, Aabenraa, Denmark

## Abstract

**Introduction:**

People with severe mental illness face markedly reduced life expectancy, largely attributable to comorbid physical illness and unhealthy lifestyle behaviors including smoking, poor diet, harmful alcohol use, and physical inactivity (SNAP factors). Despite international guidelines, psychiatric care often falls short in systematically addressing these risks.

**Objectives:**

To examine the degree of agreement between psychiatric inpatients and medical consultants regarding perceived needs for lifestyle interventions and somatic examinations/medical treatments.

**Methods:**

A prospective interventional study was conducted in the Region of Southern Denmark. A total of 231 psychiatric inpatients were consecutively recruited across seven specialised wards and assessed by 12 internal medicine consultants. Patients underwent structured medical consultations, physical examination, paraclinical tests, and a lifestyle questionnaire. Agreement between patient and consultant evaluations of intervention needs for SNAP factors and screening for diabetes, hypertension, and pulmonary disease was analysed using McNemar’s test, Cohen’s kappa, and subgroup comparisons.

**Results:**

The mean patient age was 49.4 years, with 49.8% female participants; 73.1% had at least one somatic comorbidity, and >90% presented with one or more SNAP factors. Agreement on lifestyle intervention needs was high for diet (83.7%, κ = 0.64) and physical activity (87.4%, κ = 0.58), but lower for smoking cessation (70.9%, κ = 0.61). Agreement on somatic examinations was near complete: diabetes (97.7%, κ = 0.88), hypertension (100%, κ = 1.0), and pulmonary disease (96.7%, κ = 0.89). Disagreement increased with the number of SNAP factors (p < 0.001), while younger patients were less likely to accept physical activity recommendations (p = 0.037).

**Conclusions:**

Although patient–physician agreement on somatic health needs is strong, notable discrepancies persist regarding lifestyle changes, particularly smoking cessation. Integrated, age-sensitive strategies, involving close collaboration between medical consultants and mental health professionals, are essential to improve physical health outcomes in psychiatric populations.

**Disclosure of Interest:**

None Declared

